# Partnership, sexuality, and fertility-related communication: findings from a register-based study among long-term hematological cancer survivors

**DOI:** 10.1007/s00520-022-07495-4

**Published:** 2022-12-14

**Authors:** Britta Lohmann, Katharina Kuba, Heide Götze, Anja Mehnert-Theuerkauf, Svenja Heyne, Peter Esser

**Affiliations:** Department of Medical Psychology and Medical Sociology, University Leipzig, Philipp-Rosenthal-Straße 55, 04103 Leipzig, Germany

**Keywords:** Sexuality, Partnership, Cancer survivorship, Hematological cancer, Quality of life

## Abstract

**Purpose:**

Even though the number of hematological cancer survivors suffering from long-term and late consequences of their disease is growing, knowledge about their situation regarding partnership, sexuality, and fertility-related communication is sparse to date.

**Methods:**

We recruited survivors of hematological malignancies (≥ 3 years after diagnosis) from two cancer registries in Germany. We applied validated instruments and study-specific items on satisfaction with partnership, sexual functioning, and fertility-related communication with physicians. We provided descriptive statistics and conducted multiple regression analyses to identify associations of the outcomes with patient factors and well-being (anxiety, depression, and quality of life).

**Results:**

Of 2001 eligible survivors, 922 (46%) participated. Fifty-seven percent were male, and the mean age was 64 years. Ninety percent and 60% reported to be satisfied with their partnership and sexual life, respectively. However, 81% and 86% reported being sexually impaired by physical or mental symptoms, respectively. Seventy-four percent of those with incomplete family planning had a fertility-related conversation with a physician. Female gender (*p* < .05, Beta =  − .09), older age (*p* < .01, Beta = .10), and chemotherapy (*p* < .01, Beta = .10) were associated with less sexual pleasure caused by physical impairment. Satisfaction with partnership (*p* < .001, Beta = .22), satisfaction with sexual life (*p* < .001, Beta = .28), and conversation about fertility (*p* < .05, Beta = .26) were associated with better quality of life.

**Conclusion:**

Even though long-term survivors seem to be generally satisfied with their partnership and sexual life, they may suffer from specific impairments. Our findings need to be verified in longitudinal studies.

**Supplementary Information:**

The online version contains supplementary material available at 10.1007/s00520-022-07495-4.

## Introduction

Both number of patients with hematological malignancies and respective survival rates are increasing [1, 2] which lead to a growing number of survivors who often experience physical and psychosocial long-term consequences of their disease and treatment [3, 4]. Whereas certain psychosocial aspects such as depression or quality of life have been focused on in plenty of studies [5–7], research on the possible consequences of impaired partnership, sexuality, and fertility among hematological cancer patients is sparse [8–10]. This seems problematic given that this specific patient group is particularly vulnerable to impairments in sexuality due to invasive treatments such as radiotherapy, chemotherapy [11], or hematopoietic stem cell transplantation (HSCT) [12]. Among the latter subgroup, infertility rates reach up to 75% [12].

Structure of interpersonal relationships and partnership play a crucial role in the overall course of cancer treatment and its coping [13]. Previous findings on partnership were mostly based on patients with solid tumors [14]. For example, a study among 282 breast cancer survivors found that 42% of the couples reported getting closer to each other; negative consequences were only reported in few cases; i.e., some couples (1%) or one partner (6%) reported to be more distanced to each other after the disease [15]. A study among 209 patients across different tumor sites showed that negative changes in quality of the partnership were associated with a lower quality of life and a higher risk for depression or anxiety [8]. The association of satisfaction with partnership with sociodemographic variables among cancer survivors is poorly understood. Nevertheless, a study from the general population including 1009 couples showed that men reported a higher satisfaction with partnership than women [16].

Even though studies on sexuality among hematological cancer patients exist, these are often restricted to certain subgroups: One study investigated patients with HSCT and found them to be less sexually active and to experience impaired sexual function in almost half of the sample [17]. Another study investigated 1972 patients with myeloproliferative neoplasms (MPN) and showed elevated levels of sexual dysfunction compared to controls as well as significant associations of sexual dysfunction with lower quality of life and higher levels of depression and anxiety [18]. However, patients with MPN are mostly chronic-phase patients and thus have a very specific course of disease [19], which may not be generalizable to other groups of hematological cancer. Nevertheless, a study among 4955 persons from the general population pointed to a similar result showing that physical and mental illnesses were associated with higher impairment in sexuality [20]. Another study among the general population showed an association of sexual satisfaction with gender reporting that women were more satisfied with their sexuality than men [16]. These findings, however, remain to be verified in the group of hematological cancer survivors.

Regarding fertility, a study among 878 cancer survivors showed that distress related to infertility correlated with low quality of life and psychological distress [21]. Accordingly, a study among 149 cancer survivors revealed that more than 50% of patients rated it important to discuss this aspect with the physicians, particularly those who had not completed their family planning [22]. As with partnership issues, however, most of these existing studies did not include hematological cancer types [21, 22], and thus the relevance of this topic among hematological patients remains unclear.

Given the research gaps outlined above, we used a register-based study to investigate these topics among survivors of hematological malignancies. In detail, we (i) provided the frequency of concerns on partnership and sexuality as well as fertility-related communication, (ii) tested for associations of the three outcomes with sociodemographic and medical factors, and (iii) investigated the practical relevance of the three outcomes by investigating their relationship with emotional functioning and quality of life. The findings will provide novel results regarding the relevance of these aspects among hematological cancer survivors and generate first hypotheses to identify particularly affected subgroups in order to tailor psycho-oncological programs.

## Methods

### Sample and procedure

In this cross-sectional study, we enrolled 2001 hematological cancer survivors (ICD-10: C81-C96) between the age of 18 years at time of diagnosis and 85 years at time of assessment. All data were collected between June 2015 and August 2017. Patients were recruited using two German cancer registries, i.e., the Clinical Cancer Registry of the city of Leipzig and the Epidemiologic Cancer Registry of the Federal State of Schleswig–Holstein. Eligible patients were contacted by mail. Upon agreement, participants filled in the declaration of consent and the questionnaire and sent these documents back in a postage-paid envelope. Alternatively, patients were able to participate online using the software LimeSurvey [23]. The study was approved by the ethics committee of the Medical Faculty at the University of Leipzig (file number: 292–15–24,082,015).

### Measures

#### Satisfaction with partnership

Satisfaction with partnership was measured with item 10 of the validated short form of the German questionnaire on partnership, the PFB-K [24, 25]. The item ranges from “very unsatisfied” (0) to “very satisfied” (5) on a 6-point Likert scale. If the item was not applicable, patients could select “I don’t live in a partnership” (6).

#### Satisfaction with sexuality

Given the lack of validated questionnaires in German language, satisfaction with sexuality was assessed by five internally developed items. In detail, participants were explicitly asked to estimate their level of satisfaction with their attractiveness and sexual life on a 5-point Likert scale ranging from “extremely dissatisfied” (0) to “extremely satisfied” (4). With two further items, patients reported how frequently their sexual pleasure was impaired by physical/mental strain, on a scale ranging from “never” (0) to “always” (4). The fifth item asked patients to compare their satisfaction with the current sexual life with their sexual life pre-diagnosis on a 5-point Likert scale ranging from “much worse” (0) to “much better” (4).

#### Fertility-related communication

For this outcome, we used two items which had been developed and successfully applied in a previous study on cancer survivors [22]. Using a binary response option (“yes” = 1, “no” = 0), these items assessed whether family planning was completed at time of diagnosis and whether potential negative effects of the cancer treatment on fertility have been discussed with a physician before treatment.

#### Quality of life

Quality of life was assessed using the validated German version of the European Organization for Research and Treatment of Cancer Quality of Life Questionnaire (EORTC-QLQ-C30) [26, 27]. In detail, we used the global quality of life scale (items 29 and 30), which are rated on a 7-point Likert scale ranging from “very poor” (1) to “excellent” (7). We calculated the sum score, with higher values indicating a higher quality of life.

#### Depressive symptomatology

Depressive symptomatology was assessed using the validated German version of the Patient Health Questionnaire [28, 29]. It assesses the frequency of the nine core symptoms of major depression according to DSM-IV criteria during the last 2 weeks using a 4-point Likert scale ranging from “not at all” (0) to “almost every day” (3). We calculated the sum score, with higher scores indicating higher depressive symptomatology.

#### Anxious symptomatology

Anxious symptomatology was assessed using the validated German version of the Generalized Anxiety Disorder screener [30, 31]. It assesses the frequency of the seven core symptoms of anxiety according to DSM-IV criteria during the last 2 weeks using a 4-point Likert scale ranging from “not at all” (0) to “almost every day” (3). We calculated the sum score, with higher values indicating higher anxious symptomatology.

#### Sociodemographic and medical data

Gender, age, diagnosis, and date of diagnosis were obtained from the cancer registries. Other sociodemographic and medical data were gathered via patient self-report.

### Statistical analyses

We applied descriptive statistics (percentages and means) to provide sample characteristics. Responders were compared to non-responders via Mann–Whitney *U* test (age and time since diagnosis) and chi-square test (gender and type of diagnosis).

To improve interpretability of the evaluation on partnership, sexuality, and fertility, we categorized each of the outcomes to form meaningful categories for all results referring to the first research question. Subsequently, these categories were descriptively analyzed by presenting raw values and percentages (for details, see Table 2).

To identify sociodemographic and medical factors that are associated with sexuality and partnership, we conducted multiple linear regression analyses. We selected factors proven to be relevant in previous research [11, 12, 32–35], i.e., age (years), gender (male/female), time since diagnosis (years), remission status (not in remission/in remission), chemotherapy (no/yes), and radiotherapy (no/yes). For each outcome variable, a separate model including all sociodemographic and medical factors was run. Standardized regression coefficients were provided to ensure comparability of the factors regarding significance and size.

To investigate the association of the items on partnership, sexuality, and fertility with well-being, we applied separate univariate regression analyses to assess their respective relationship with global quality of life, and depressive and anxious symptomatology. The robustness of these regressions (unconditional models) was checked by re-running the analyses controlled for the sociodemographic and medical variables defined above (conditional models).

Adjusted *R*^2^ was reported as effect size, indicating magnitude of explained variance in the outcome. The alpha level was set at 0.05. Sum scores were only computed if more than 50% of the respective scale were available. Listwise deletion was applied for the regression analyses. Missing values of the outcomes ranged between 3 (conversation with physician concerning fertility) and 22% (impairment of sexual joy by mental strain). Analyses were performed using SPSS 26 (2019, IBM Corporation, Armonk, USA).

## Results

Of 2001 eligible survivors that could be reached, 922 participated in the study (response rate: 46%). Participants were slightly younger (*p* = 0.001; *x*_diff_ = 1.5 years) compared to non-responders, but did not significantly differ in gender, type of diagnosis, or time since diagnosis (Table 1).

**Table 1:** Patient characteristics

	Participants *N* = 922	Non-participants^a^ *N* = 1079	*p*^b^
	*N* (valid %)	*N* (valid %)	
Sociodemographic			
Gender (male)	527 (57)	602 (56)	.539
Age, *M (SD)*	63.9 (13.4)	65.5 (14.1)	.001
Currently living in partnership	734 (80)		
Medical			
Cancer type according to ICD-10			.215
Hodgkin lymphoma (C81)	101 (11)	117 (11)	
Follicular lymphoma (C82)	123 (13)	168 (16)	
Non-follicular lymphoma (C83)^d^	247 (27)	319 (30)	
Other non-Hodgkin lymphoma (C85)	59 (6)	62 (6)	
MM/MPCN (C90)^e^	118 (13)	126 (12)	
Lymphoid leukemia (C91)^f^	140 (15)	135 (13)	
Myeloid leukemia (C92)^g^	95 (10)	93 (9)	
Others	39 (4)	59 (6)	
Years since diagnosis, *M (SD)*	9.1 (4.2)	8.9 (4.5)	.249
2.5–5.9 years (cohort 1)	262 (28)		
6.0–8.9 years (cohort 2)	222 (24)		
9.0–11.9 years (cohort 3)	179 (19)		
≥ 12 years (cohort 4)	257 (28)		
In remission	634 (73)		
History of relapse	201 (24)		
Second tumor^h^	155 (17)		
Treatment^i^			
Chemotherapy	722 (79)		
Radiotherapy	391 (43)		
Anti-body therapy	198 (22)		
Surgery	151 (17)		
SCT	244 (27)		

*MM/MPCN* multiple myeloma and malignant plasma cell neoplasms, *SCT* autologous and/or allogeneic stem cell transplantation

^a^All reachable patients who declined or did not respond

^b^Gender/cancer type: chi-square test; age/time since diagnosis: Mann–Whitney *U* test

^d^Mostly B cell lymphoma (52%)

^e^Mostly multiple myeloma (95%)

^f^Mostly chronic (76%)

^g^Mostly acute (69%)

^h^Before or after hematological malignancy

^i^All treatment related to the hematological malignancy; combinations possible

Ninety percent reported to be satisfied with their partnership, and more than half of the patients reported to be satisfied with their sexual life (Table 2). Nevertheless, 4 out of 5 patients also reported their sexual pleasure to be frequently impaired by physical and mental symptoms; furthermore, half of the patients described their sexual life to be worse compared to pre-diagnosis. Forty percent reported to be satisfied with their attractiveness, a third reported to be unsatisfied. Almost 75% of those whose family planning was not completed confirmed to have had a conversation with a physician about fertility issues.

**Table 2:** Satisfaction with partnership and sexuality as well as fertility-related communication

		*N*	Valid %
Partnership			
Satisfaction with partnership^a^	Unsatisfied	70	10
	Satisfied	641	90
Sexuality			
Satisfaction with sexual life^b^	Unsatisfied	174	21
	Satisfied	496	60
	Neutral	163	20
Comparison to pre-diagnosis^c^	Worse	372	51
	Better	318	43
	Equal	43	6
Satisfaction with attractiveness^b^	Unsatisfied	271	35
	Satisfied	314	40
	Neutral	163	20
Impairment by physical symptoms^d^	Frequently	588	81
	Not frequently	136	19
Impairment by mental symptoms^d^	Frequently	622	86
	Not frequently	99	14
Fertility			
Conversation with physician^e^	No	26	26
	Yes	74	74

Percentages may not add up to 100 due to rounding

^a^Only patients living in a partnership were included (*n* = 711); *unsatisfied*** = **“very unsatisfied,” “unsatisfied,” “rather unsatisfied”; *satisfied* = “rather satisfied,” “satisfied,” “very satisfied”

^b^*Unsatisfied* = “very unsatisfied,” “unsatisfied”; *satisfied* = “satisfied,” “very satisfied”; *neutral* = “neither nor”

^c^*Worse* = “much worse,” “rather worse”; *better* = “rather better,” “much better”

^d^*Frequently* = “almost always,” “always”; *not frequently* = “never,” “seldom,” “sometimes”

^e^Only patients whose family planning was not completed were included (*n* = 100)

Neither sociodemographic nor medical factors were significantly associated with satisfaction with partnership (Table 3). For the items on sexuality, however, a more differentiated picture emerged: For example, gender and remission status were associated with all variables, whereas radiotherapy was not associated with any of the sexuality items. Furthermore, all associations were small, with all factors together explaining only up to 5.1% of variance.

**Table 3:** Relationship of satisfaction with partnership/sexuality with sociodemographic and medical factors

	Model summary^a^		Beta^a^					
	*N*	*R*^***2***^	*Age*	*Gender*^***b***^	*Time since diagnosis*	*Remission status*^***c***^	*Chemotherapy*^***d***^	*Radiotherapy*^***e***^
Partnership								
Satisfaction with partnership	675	0	.042	.001	− .014	.043	− .073	− .023
Sexuality								
Satisfaction with sexual life	743	.029	− .079*	− .109**	− .030	.104**	− .074	− .044
Comparison to pre-diagnosis^f^	704	.051	− .149***	− .086*	.038	.120**	− .126**	− .052
Satisfaction with attractiveness	788	.029	.064	.114**	.027	.086*	− .109**	− .046
Impairment by physical symptoms	695	.046	.102**	− .090*	− .074	− .165***	.099**	− .002
Impairment by mental symptoms	693	.035	.089*	− .100**	− .096*	− .125**	.038	− .011

*Beta* standardized regression coefficient, *R*^2^ adjusted *R*-squared

^*^p < .05; **p < .01; ***p < .001

^a^In each analysis, all sociodemographic and medical factors were included

^b^0 = female, 1 = male

^c^0 = not in remission, 1 = in remission

^d^0 = no chemotherapy, 1 = chemotherapy

^e^0 = no radiotherapy, 1 = radiotherapy

^f^0 = much worse to 4 = much better

Satisfaction with partnership and sexual life as well as fertility-related conversations were associated with well-being: All variables concerning partnership and sexuality were associated with higher quality of life, and lower levels of depressive and anxious symptoms. Similarly, having had a fertility-related conversation with a physician was associated with a higher quality of life and less depressive symptomatology (Table 4). The strongest associations with well-being were found in the relationship between satisfaction with attractiveness and quality of life/depressive symptomatology.

**Table 4:** Relationship of satisfaction with partnership and sexuality as well as fertility-related conversation with well-being

	Quality of life			Anxious symptoms			Depressive symptoms		
	*N*	Beta	*R*^2^	*N*	Beta	*R*^2^	*N*	Beta	*R*^2^
Partnership									
Satisfaction with partnership	662	.235***	.054	663	− .241***	.056	665	− .247***	.059
Sexuality									
Satisfaction with sexual life	730	.300***	.089	731	− .186***	.033	733	− .246***	.059
Comparison to pre-diagnosis^a^	693	.328***	.106	692	− .186***	.033	694	− .230***	.051
Satisfaction with attractiveness	772	.442***	.195	775	− .326***	.105	777	− .442***	.194
Impairment by physical symptoms	685	− .373***	.138	686	.200***	.038	687	.271***	.072
Impairment by mental symptoms	683	− .356***	.125	684	.325***	.104	685	.346***	.119
Fertility									
Conversation with physician^b^	96	.295**	.077	96	− .116	.033	96	− .203*	.031

*Beta* standardized regression coefficient, *R*^2^ adjusted *R*-squared

^a^0 = much worse to 4 = much better

^b^Among patients whose family planning was not completed

^*^p < .05; **p < .01; ***p < .001

Except for one association (fertility-related conversation with depressive symptomatology), all of these univariate associations remained significant in the controlled models after including sociodemographic and medical variables (Table S1).

## Discussion

### Main findings

This study among survivors of hematological malignancies showed that the majority reported to be satisfied with their partnership and sexual life despite perceived impairments in their sexual pleasure. Fertility issues were mostly discussed with the physicians. Associated factors could be identified for variables on sexuality, but not for partnership. All the three outcomes were associated with well-being.

### Integration into previous research

Regarding partnership, Geue et al. examined 99 patients across cancer types with a mean of 30 months post-diagnosis and found that 76% rated their quality in partnership as high [36]. Our findings largely confirm this finding, with 90% being satisfied with their partnership. Using our sample of long-term hematological cancer survivors with a mean of 9 years after diagnosis, we could extend previous knowledge showing high partnership quality in long-term cancer patients. However, we did not assess whether patients had the same partner across the whole illness trajectory. Therefore, surveys to assess separations across the course of the disease or other changes in partnership status should be applied in the future.

We did not find any associations of sociodemographic or medical factors with satisfaction with partnership, which corresponds to another study testing the association between satisfaction with partnership and gender among non-Hodgkin lymphoma survivors [35]. Among 1009 couples from the general population, however, men were shown to be more satisfied with their relationship than women [16]. Given the discrepancies and the paucity of relevant research, future studies on this issue are needed. A possible explanation for the lack of associations of satisfaction with partnership with most other patient factors could be that satisfaction with partnership may be more dependent on stable personality traits by both the partners and the patients [37]. Moreover, we found that satisfaction with partnership was significantly associated with higher levels of well-being. Previous research on the relationship between satisfaction with partnership and well-being among cancer patients is sparse. However, our findings largely correspond to a review among 73 studies (with only one including cancer patients), which found an association between dissatisfaction in the partnership and anxious symptomatology [38]. Our data could show the importance of this issue among the vulnerable group of cancer survivors.

With respect to sexuality, Geyer et al. examined 1971 patients with myeloproliferative neoplasms and found that sexual dysfunction correlated with lower levels of quality of life and higher levels of distress [18]. This result corresponds with our study, in which we found an association with higher levels of depression and anxiety and lower quality of life. With respect to associated factors, previous studies showed inconsistent findings concerning age and gender [16, 18, 20, 39, 40]. We found that older age correlated with a lower satisfaction with sexual life and higher levels of sexual impairment. Furthermore, female gender correlated with a higher satisfaction with sexual life, but also more impairment by physical and mental symptoms. Further studies are needed, e.g., whether female patients have more effective ways than men to cope with impairments in order to maintain a high satisfaction with their sexual life.

Regarding satisfaction with attractiveness, patients were less satisfied if they had received chemotherapy or were female. The gender effect corresponds to a study among 50 hematological cancer survivors demonstrating that female patients were more likely to have an impaired body image [41]. Also consistent to our findings, a study including 549 women with breast cancer showed that hair loss from chemotherapy was associated with elevated levels of body image problems [42]. Given the aforementioned study, future research among cancer survivors may examine the occurrence of visible body changes as possible moderating or mediating factors to explain associations of negative perceptions of attractiveness with type of treatment.

With respect to fertility, a study among 149 cancer patients across tumor sites found that more than two thirds of the patients with uncompleted family planning talked to their oncologist about fertility [22]. This is in line with our findings, with more than two-thirds of those with uncompleted family planning having communicated with their physician about fertility-related issues. Even though it could not be identified whether the patients or the physicians initiated these conversations, it implies that this issue is frequently addressed within the oncological care. Nevertheless, about one-third did not discuss this important aspect, which may have detrimental consequences. Our finding on an association between such conversations and a higher well-being is consistent with a study of 918 women including 587 with hematological malignancies [43] and thus verifies the relevance on this issue among hematological cancer patients.

### Clinical implications

We found that patients rated their satisfaction with sexual life overall positive, but also reported high perceived impairments since diagnosis. Therefore, clinicians should not only rely on general questions on satisfaction but should ask more for specific problems in sexual life to offer adequate symptom management. In addition, physicians should communicate side effects prior to initiating treatment to enable informed treatment decisions and provide patients with a better sense of control. We also found that partnership, sexuality, and fertility issues are associated with well-being. This in turn demonstrates that these issues must not be neglected in routine care but should be addressed even though patients may not initiate such topics. Female gender, older age, being non-remitted, and having received chemotherapy were associated with lower satisfaction with sexual life and, with the exception of female gender, also with lower satisfaction with partnership—therefore, clinicians may use these findings to pay specific attention to these patient groups.

### Strengths

To our knowledge, this study is one of the few addressing partnership, sexuality, and fertility among hematological cancer survivors and thus may provide valuable information to establish hypotheses in this neglected area of research. The large sample size contributes to the robustness of the results and the sample including patients up to 26 years after diagnosis (with a mean of 9 years post-diagnosis) enabled us to add new findings in the long-term survivorship phase. The register-based approach ensured valid data on central sociodemographic and medical patient characteristics. We also used effect sizes to estimate the relevance of significant findings.

### Limitations

Our cross-sectional design did not allow to interpret the findings in a causal manner. Nevertheless, we could establish important hypotheses to be verified in future longitudinal studies. Given the lack of available instruments, our outcomes concerning sexuality were internally developed and thus not validated. Nevertheless, we note that the questions were developed within extensive discussions and were taken from other studies wherever possible. Future research should develop a comprehensive questionnaire to ensure validity and comparability across studies. Even though central patient characteristics could be obtained from the registry, some variables such as treatment and disease status were obtained via self-report and thus have limited validity. Furthermore, we did not have information on disease stage and thus were not able to control for this issue in our regression analysis. Moreover, we had a relatively low response rate (46%), which bears the risk of a sample bias. Nevertheless, responder analyses showed that responders and non-responders did not meaningfully differ in central characteristics. Missing values in our outcomes reached up to 22%. A possible reason might be the intimacy and anticipated stigmatization of the topics which may have hindered patients to answer the questions. Assuming that patients with high distress in these topics may have felt less comfortable to answer these questions, our results may have been biased towards a more positive evaluation. Future studies may use a small introduction text to validate the difficulty to report about this subject together with the importance to do so. Regarding a fertility-related discussion, we could not identify whether these conversations were initiated by the patients or by physicians. Future studies need to investigate this in more detail to draw clear conclusions.

## Conclusion

The majority in our sample of long-term survivors of hematological malignancies were satisfied with their partnership and sexual life, but many experienced specific impairments in their sexual pleasure. Together with associations of these issues with well-being, we conclude that more attention should be paid to sexual health in oncological settings. Female patients, those who are older or treated with chemotherapy, may be particularly impaired and thus need to be focused on. To draw causal conclusions, our findings need to be verified in longitudinal studies.

## Supplementary Information

Below is the link to the electronic supplementary material.

Supplementary file1 (15.6 KB)

## Data Availability

The data and code used for this publication are available from the corresponding author on request.
