# Supplementary material for: Partnership, sexuality, and fertility-related communication: findings from a register-based study among long-term hematological cancer survivors
Source: Support Care Cancer. 2022 Dec 14;31(1):26. doi: 10.1007/s00520-022-07495-4 (PMC9747843; doi:10.1007/s00520-022-07495-4)
Supplement: Supplementary file 1 — Supplementary file1 (15.6 KB) [file 520_2022_7495_MOESM1_ESM.docx]

| **Table S1.** *Relationship of satisfaction with partnership and sexuality as well as fertility-related conversation with well-being, controlled for sociodemographic and medical variables^a^* | | | | | | |
| --- | --- | --- | --- | --- | --- | --- |
|  | **Quality of Life** | | **Anxious symptoms** | | **Depressive symptoms** | |
|  | **N** | **Beta** | **N** | **Beta** | **N** | **Beta** |
| **Partnership** | | | | | | |
| Satisfaction with partnership | 662 | .222*** | 663 | -.226*** | 665 | -.233*** |
| **Sexuality** | | | | | | |
| Satisfaction with sexual life | 730 | .278*** | 731 | -.211*** | 733 | -.261*** |
| Comparison to pre-diagnosis^b^ | 693 | .302*** | 692 | -.216*** | 694 | -.250*** |
| Satisfaction with attractiveness | 772 | .425*** | 775 | -.277*** | 777 | -.403*** |
| Impairment by physical symptoms | 685 | -.341*** | 686 | .191*** | 687 | .258*** |
| Impairment by mental symptoms | 683 | -.333*** | 684 | .356*** | 685 | .337*** |
| **Fertility** | | | | | | |
| Conversation with physician^c^ | 96 | .260* | 96 | -.096 | 96 | -.208 |
| **Beta**, standardized regression coefficient; * p < .05, ** p < .01, *** p < .001;  ^a^ age (years), gender (male/female), time since diagnosis (years), remission status (not in remission/in remission), chemotherapy (no/yes) and radiotherapy (no/yes); ^b^ 0=much worse to 4=much better; ^c^ among patients whose family planning was not completed; | | | | | | |
